# Visual performance of painting colors based on psychological factors

**DOI:** 10.3389/fpsyg.2022.966571

**Published:** 2022-09-29

**Authors:** Chenchen Yao, Tian Tian, Cai Gao, Shuangping Zhao, Qingyan Liu

**Affiliations:** ^1^Dongguk University, Art Academy, Seoul, South Korea; ^2^College of Fine Arts, Shandong Normal University, Jinan, China

**Keywords:** psychological factors, painting color, visual performance, color psychology, variance

## Abstract

Humans have been exploring colors since ancient times, but relatively complete color systems appeared one after another in the twentieth century. Even without language and other information exchanges, colors can still convey information and stimulate emotions. Therefore, color can have both physical and psychological effects on people. In this context, this paper studies the visual representation of painting colors based on psychological factors. The article studies the theory of personality traits and introduces the related content of visual psychology. To explore the relationship between each variable and color psychology and the visual representation of painting colors, a binary logistic regression analysis is performed. The colors in the post-impressionist paintings of Van Gogh and Gauguin is contrasted, and experiments on psychological factors and color research is conducted. The factors that affect the color tone of the picture and the influence of psychological factors on the judgment of color brightness are investigated. Finally, the correlation analysis of personality trait dimension and irrational behavior is carried out. The experimental results of the article show that after the analysis of variance, the significance levels of regression model 1 and model 2 both reach 0.000, and the adjusted R squares are 0.319 and 0.356, respectively. In this study, regression model 2 was selected as the final model. According to Model 2, the standardized regression coefficients of agreeableness and neuroticism are 0.438 and -0.251, respectively, and the significance of the regression coefficients are 0.000 and 0.021, respectively. The research on the visual performance of painting colors based on psychological factors has been well completed.

## Introduction

Modern psychology has found in the research on the acquisition of environmental spatial information by people’s different senses that vision is the most important way to acquire, and its acceptance is far greater than that of hearing, smell, and touch. Psychology has never been a unified discipline. In the words of the philosopher of science Kuhn, psychology lacked a stable “paradigm” and never had a theoretical foundation acceptable to a disciplinary community like other normative sciences. Relevant research data shows that in the first 20 s of the visual senses capturing information such as color, shape, texture and others, color occupies an absolute advantage, reaching about 80%. After 2 min, as other information is gradually paid attention, color occupies 60% of the feeling. After 5 min, the color occupies 50% of the perception and maintains this sensory ratio for a long time. It can be seen that the impression of color is rapid, profound and lasting. Psychological factors include two aspects: psychological process and personality. Mental process is composed of cognitive process, emotional process and will process. Personality includes personality tendencies and personality psychological characteristics.

The quality of color matching directly affects people’s physical emotions. High-quality color matching should convey to people a comfortable and pleasant mood, which is harmonious, beneficial, safe and sustainable. Poor color matching conveys emotions such as anxiety and unease, which are sharp, harmful and irreversible. It can be said that “color is the most emotional, it affects people’s emotional level, far beyond other factors, it gives people unlimited reverie”. The study of psychological factors is crucial to the subject of the visual representation of painting colors.

## Related work

Flora DB investigated the calibration method using non-cognitive scales between groups of students with different educational backgrounds. His calibration method was used to estimate measurement models and to detect measurement non-variance across multiple groups (that is DIF). Divergence is an indicator in stock market technical analysis, abbreviated as DIF, which is the value of the 12-day EMA minus the 26-day EMA value. The 12-day EMA is above the 26-day EMA in the ongoing rally. The positive difference (+DIF) between them is getting bigger and bigger. Conversely, in a downtrend, the divergence value may become negative (-DIF) and become larger and larger. His research aimed to explore and analyze confirmatory factors in psychological research. However, his research did not build a specific analysis method or module, and was not applicable (Flora and Flake, 2017). Karpinski R I surveyed members of Mensa Ltd. (*n* = 3,715) in the United States to explore psychoneuroimmune (PNI) processes in individuals at or above the 98th percentile of intelligence. Participants were asked to self-report diagnosed and/or suspected mood and anxiety disorders, attention deficit hyperactivity disorder (ADHD), autism spectrum disorder (ASD), as well as the prevalence of physiological conditions including environmental and food allergies, asthma and autoimmune diseases. He argued that those with high intellectual abilities (superbrains) were hyperexcitable in various domains, which might predispose them to certain psychological disorders (Karpinski et al., 2017). Bessaha (2017) believed that researchers and practitioners could confidently use K6 to screen for symptoms of psychological distress in emerging adult populations. He found that each model exhibited a good fit, with significant loadings for each factor. The second-order two-factor model and the two-factor model are equivalent and fit better than the one-factor model. Sviderskiy et al. (2021) worked to improve the quality of medium-range rockets and spacecraft manufactured by the joint-stock company Rocket Space Center Progress. His research reflected findings on the psychological and emotional state of Earth-sensing rocket and spacecraft assembly workers and their impact on labor process efficiency. But his research did not mention that communication control and low stress tolerance among employees can lead to low performance.

Stepanova (2019) showed methods for analyzing color composition: how to extract color palettes from painted images, and how to measure color characteristics. He believed that paintings with high color diversity were of higher value than equivalent works of art in a monochromatic style. But his research did not take into account the artistic value in paintings. Xiang and Wu (2017) conducted research on Chinese folk New Year pictures. He believed that, in essence, Chinese folk New Year pictures are a visual expression, and their nature of pasting at home made them a visual encirclement for Chinese people. This visual encirclement gradually expands in the home space, which forms a spiritual home with different cultural spaces, living environments, image schemas, and secular religious beliefs. Casas (2018) conducted research on Noble Patents. He believed that the customs and aesthetics of the nobility were intended to be imitated in this social class in order to distinguish it from the rest of the society. Among them, there are illustrated examples reflecting the evolution of large-format painting. Kim et al. (2019) analyzed the material and composition features of oriental paintings, and proposed a feature extraction method suitable for oriental paintings. An oriental painting recommendation method is also suggested to provide users with customized digital content to support health. However, his research did not mention the contrast between Eastern and Western paintings, as well as material properties and compositional properties, which have certain material limitations.

## Psychological factors and methods of color expression

### Personality trait theory

Personality is a unique behavioral pattern and way of thinking that determines a person’s adaptation to the environment. It is the sum of the relatively stable psychological characteristics of an individual with a certain tendency, including both psychological characteristics and external behavioral characteristics (Kelif, 2018). Personality trait theories mainly include Allport’s personality trait theory, Cartel’s personality factor theory, Eysenck’s personality structure theory, Roth’s point of control theory, Jung’s psychological type theory and “Big Five” personality trait theory (Chen, 2017). The Big Five (OCEAN), also known as the Ocean of Personalities, can be assessed by NEO-PI-R. The following only focuses on the theory of the “Big Five” traits that are directly related to this study.

Neuroticism: it is used to measure the degree of emotional stability, reflecting the tendency to experience negative emotions and emotional instability. High scorers are prone to tension, depression, anxiety, impulsiveness and insecurity, poor emotional regulation and coping abilities, and poor thinking, decision-making, and ability to effectively deal with external pressures (Dorot, 2020). The key to the formation of neuroticism is two points: one is the neurotic character; the other is determined by the neurotic character, which focusing too much on perception or trying to eliminate symptoms. As a result, the patient’s life deviates from the norm and falls into a vicious circle.

Extraversion: it is used to measure an individual’s ability to respond to interpersonal relationships and external stimuli and feel pleasure from them. High scores indicate that individuals are more confident, social, optimistic, positive, energetic, easy to feel various positive emotions; assertive, dominant, persuasive; decision-making without hesitation, fast-paced, thrill-seeking, and adventurous (Genç and Öner, 2018).

Openness: it is used to measure an individual’s cognitive style, receptivity and curiosity toward unfamiliar situations and new things. A high score indicates that an individual can break through tradition, be highly curious, pursue curious, novel, non-traditional and creative things, be open-minded, prefer abstract thinking, and be imaginative (Oloidi, 2017).

Agreeableness: it is used to measure an individual’s attitude and communication style toward others in interpersonal communication, and the degree of cooperation and interpersonal harmony in teamwork. High scorers tend to be close to others, trust others, be tolerant, have compassion, tend to think and act from the perspective of others, prioritize the interests of others, be helpful, cooperative, empathetic, and optimistic about human nature (Yu and Xiaoting, 2018).

Conscientiousness: it is used to measure a person’s will and determination, and the degree to which they control, manage, and regulate their own behavior. High scorers tend to behave according to established rules, make decisions based on standards and basis, are strong-willed, determined, organized, reliable, punctual, methodical, cautious, and logical, task-focused and meticulous (Karabas, 2019).

### Color design

Color is transmitted through feeling. When people see different colors, they have different psychological feelings. Even colors of the same hue with different lightness give people different feelings. For example, bright red gives a warm feeling, and dark red gives a bloody feeling. In addition, it is associative and symbolic. Green conjures images of military uniforms, plants, and bright yellow conjures things up steaming and delicious. The combination of colors is also very important in the design of paintings. For example, the combination of black and red gives people a bloody, dark and uncomfortable feeling, and the combination of red and yellow gives people a warm, sunny and comfortable feeling (Vries et al., 2020). In the painting design, the audience’s preference for color and the psychological feelings brought by it should be considered. Compared with young people who prefer bright and saturated colors, older people prefer calm and steady colors. In the collocation of different colors, it is necessary to achieve coordination and visual balance. The primary color of the material for design and matching can be tried, such as wood. Its own log color gives people a rustic, natural, and environmentally friendly feeling, and no further processing is required. Different colors can also be used to separate different functions (Drew, 2020).

### Visual psychology

Visual psychology is a branch of psychology. It is widely used in the field of art design and creation, and is used in planes, indoor and outdoor spaces, film and television, sculpture and other works. The domestic research on visual psychology includes “Art Effect and Visual Psychology.” In this work, the relationship between visual psychological effect and form is explained in an easy-to-understand way through the fusion of practical case analysis and theory. Its point of view is clear, and the diagram is also different from other common theories (Lowenthal et al., 2020).

Since the formation of visual psychology is not long, the research on its application in display design is also very rare (Evba et al., 2020). It can be seen from the data that some papers and teaching materials related to display space design roughly mention the knowledge of visual psychology, but the depth is not enough (Chaika, 2020). By starting from the related theories of display design and visual psychology, it studies the principle of the role of visual psychology in the relevant elements of display design. And from the two aspects of how to make a design that is more in line with the psychological demands of visitors with the guidance of the relevant theories of visual psychology, and from the gradually increasing number of exhibition activities and the importance of visual psychology, the presentation method of the exhibition space and the information transmission method are expounded in the aspect of visual psychology by studying the initial reactions and needs of people’s psychological world.

The Gestalt principle advocates the study of direct experience (that is, consciousness) and behavior, emphasizes the integrity of experience and behavior, believes that the whole is not equal to and greater than the sum of the parts, and advocates the study of psychological phenomena with the view of the dynamic structure of the whole. The Gestalt principle breaks the limits of corners and regions, which showing obvious unconscious characteristics. There are some commonalities in human visual perception, namely the “vision principle.” These visual principles mainly have the following aspects: inspiration for the arrangement of pictures, constancy of vision, trompe l’oeil phenomenon, completeness and closure tendency, the “touch function” of visual perception, psychological balance, the influence of motor perception on multimedia display.

### Binary logistic regression resolve

The dependent variable of logistic regression can be binary or multi-category, but binary classification is more commonly used and easier to explain. Multi-category can be processed by using the softmax method. The most commonly used in practice is the binary logistic regression. Are the variables related to color psychology and the visual representation of painting colors? In order to further explore the relationship between various factors and the occurrence of financial restatement, this section intends to use the method of logistic regression to verify the research hypothesis proposed above. The specific construction steps of the logistic regression model are as follows.

Regression model:


(1) $$\text{Y}=\beta_{0}+\beta_{0}*X_{1}+\ldots +\beta_{\text{m}}*X_{\text{m}}+\text{ϵ}$$

In this Equation, Y takes the value of 0 or 1, and record Pi = P{Y = 1} = P {financial restatement occurs}, then the probability that the listed company don’t have financial restatement is 1-Pi, E(Y) = Pi. It can be seen from the linear regression Equation that, given the conditions of each variable, the conditional mathematical expectation of Y is:


(2) $$\text{E}\,\left(\text{T}/{\text{X}}_{1},\ldots ,{\text{X}}_{\text{m}}\right)=\beta_{0}+\beta_{0}*X_{1}+\ldots +\beta_{\text{m}}*X_{\text{m}}+\text{ϵ}$$

Therefore:


(3) $${\text{P}}_{\text{i}}=\beta_{0}+\beta_{0}*X_{1}+\ldots +\beta_{\text{m}}*X_{\text{m}}+\text{ϵ}$$

According to the logistic function expression:


(4) $$\text{ln}\,\frac{\text{p}}{1-\text{p}}=\text{z}$$

On this basis, the logistic regression Equation is proposed:


(5) $$\text{L}=\beta_{0}+\beta_{0}*X_{1}+\ldots +\beta_{\text{m}}*X_{\text{m}}$$

The final design of the logistic model is:


(6) $$\text{L}=\text{ln}\,\frac{\text{p}}{1-\text{p}}$$

(7) $$\text{L}=\beta_{1}*\mathrm{Motivation}+\beta_{2}*\mathrm{Character}+\beta_{3}*\mathrm{Ability}+$$

$$\beta_{4}*\mathrm{Temperament}+\beta_{5}*\mathrm{Value}+\beta_{6}*\mathrm{Experience}+\beta_{7}*\mathrm{Title}$$

$$+\beta_{8}*\mathrm{Education}+\beta_{9}*\mathrm{CPA}+\beta_{10}*\mathrm{Audit}+\beta_{11}*\mathrm{ROE}+\text{ϵ}$$

The color of the regression coefficients of each explanatory variable in the model indicates the degree of change in the logarithm of whether a color restatement occurs for each unit of change in an explanatory variable.

In PSO, the solution of each optimization problem is a bird in the search space, which can also be called a “particle.” All particles have a fitness value determined by an optimized function, and each particle also has a velocity that determines the direction and distance they fly. Then the particles follow the current optimal particle to search in the solution space.

In order to optimize the above logistic model, the basic particle swarm algorithm is used for optimization. The particle swarm algorithm has two extreme values, which are the individual extreme pbest value and the group extreme value gbest. The swarm mechanism is the global optimal solution. The basic idea of the basic particle swarm algorithm is: in each iteration, each particle continuously updates its speed and position in the solution space according to the Equation, and approaches the region where the two extreme values are located.

pbest is defined as:


(8) $$p\,b\,e\,s\,t_{1}={\left(p\,b\,e\,s\,t_{i\,1}\cdot p\,b\,e\,s\,t_{i\,2}\cdot p\,b\,e\,s\,t_{i\,3}\,\ldots \,p\,b\,e\,s\,t_{i\,N}\right)}^{T}$$

Gbest is defined as:


(9) $$g\,b\,e\,s\,t_{1}={\left(g\,b\,e\,s\,t_{i\,1}\cdot g\,b\,e\,s\,t_{i\,2}\cdot g\,b\,e\,s\,t_{i\,3}\,\ldots \,g\,b\,e\,s\,t_{i\,N}\right)}^{T}$$

Equation (10) is the update equation of particle velocity and position:


(10) $$\left\{ \begin{array}{c} V_{i\,d}^{k+1}=\forall \omega V_{i\,d}^{k}+C_{1}\times r_{1}^{k}\times \left(pbest_{i\,d}^{k}-x_{i\,d}^{k}\right)+c_{2}\times r_{2}^{k}\times \\ \left(g\,b\,e\,s\,t_{i\,d}^{k}-x_{i\,d}^{k}\right)\\ x_{i\,d}^{k+1}=x_{i\,d}^{k}+v_{i\,d}^{k+1} \end{array}\right.$$

**FIGURE 1:**
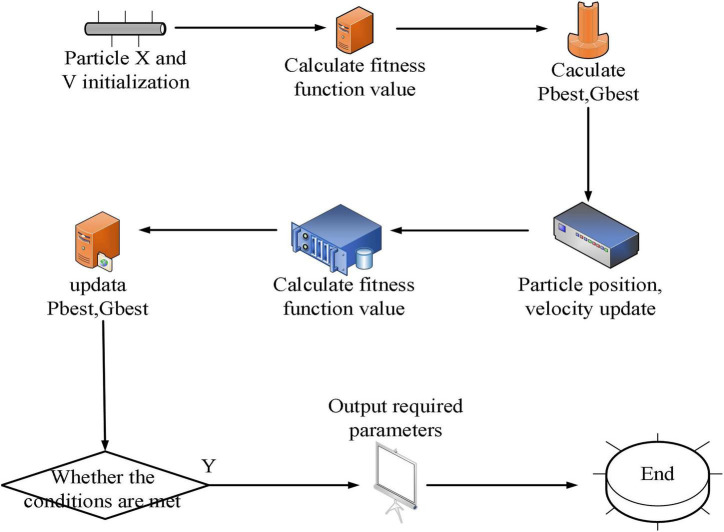
Flow chart of particle swarm algorithm.

The flow of the basic particle swarm algorithm is shown in Figure 1.

The particle determines the value of the corresponding position of the particle according to the size of the speed. The greater the speed, the greater the probability of the position being 1 is. The velocity update equation and position update equation of the binary particle swarm are:


(11) $$\left\{ \begin{array}{c} v_{i\,d}=\omega v_{i\,d}+c_{1}\times r_{1}\times \left(pbest_{i\,d}-x_{i\,d}\right)+c_{2}\times r_{2}\times \\ \left(p\,b\,e\,s\,t_{i\,d}-x_{i\,d}\right)\\ if\left(rand\left(\right)<s\left(v_{i\,d}\right)\right)thenx_{i\,d}=1,elsex_{i\,d}=0 \end{array}\right.$$

The velocity of the particle is updated according to the Sigmoid function, which is defined as Equation (12):


(12) $$\text{Sigmoid}\,\left({\text{v}}_{\text{id}}\right)=1/\left(1+{\text{e}}^{-{\text{v}}_{\text{id}}}\right)$$

In this case the logistic mapping equation can be defined as:


(13) $$X_{n+1}=X_{n}\times \mu \times \left(1-X_{n}\right)$$

The controlled logistic mapping equation is shown in Equation (14):


(14) $$X_{n+1}=X_{n}\times \mu \times \left(1-X_{n}\right)+\mu \,n$$

Finally, a set of chaotic variables is introduced into the inertia weight of the particle swarm optimization algorithm. The certainty of a dynamical system is a mathematical concept, which means that the state of the system at any time is determined by the initial state. The inertia weight equation after introduction is shown in Equation (15):


(15) $$\omega \,\left(\text{n}\right)=\omega_{\text{min}}+\left(\omega_{\text{max}}-\omega_{\text{min}}\right)\,\text{x}\,\left(\text{n}\right)$$

In this way, the search range of the algorithm and the global and local search capabilities have been greatly improved.

The article uses a multidisciplinary integration model. Multidisciplinary integration refers to the research, teaching and application of interdisciplinary and multidisciplinary interdisciplinary integration, interdisciplinary science and interdisciplinary integration.

### Color in post-impressionist painting

Post-Impressionist artists maximized the shape and color of their paintings, almost disregarding any subject matter and content, and used subjective feelings to shape objective phenomena. Color is a key component of artistic language. It carries the spirit of art and plays a vital role in painting creation, which cannot be replaced by any artistic language. Color paintings do not have the assumed quality of black and white paintings, and color paintings are more realistic than black and white paintings. In this respect, color painting not only allows people to see the shape and space, but also shows the color difference between objects. The color form can satisfy the viewer’s curiosity and at the same time be more friendly.

**FIGURE 2:**
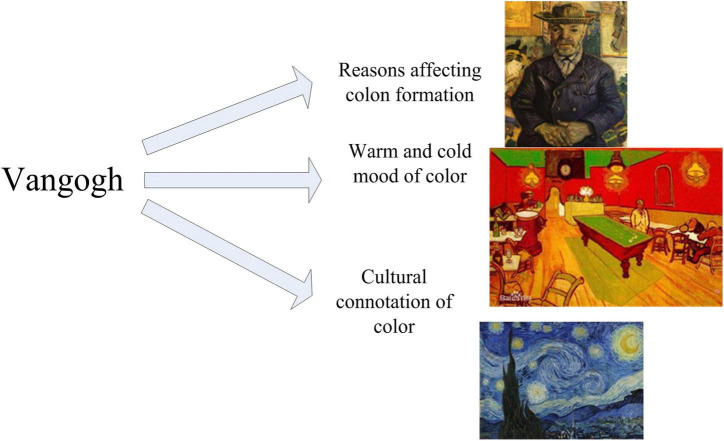
Colors in Van Gogh’s paintings.

In an oil painting, color is the most important factor. By comparing color with shape, Arnheim argues: “As a communication tool, shape is more effective than color, but the expression obtained by using color cannot be obtained by shape…The emotion conveyed by the afterglow of the setting sun and the turquoise blue of the Mediterranean is probably beyond the reach of any definite shape.” It can be seen that color is the most important painting factor. Artists in different periods have spent their whole lives exploring the expressive power of color. Post-Impressionism accomplished a complete revolution in color. The colors in Van Gogh’s paintings are shown in Figure 2.

**FIGURE 3:**
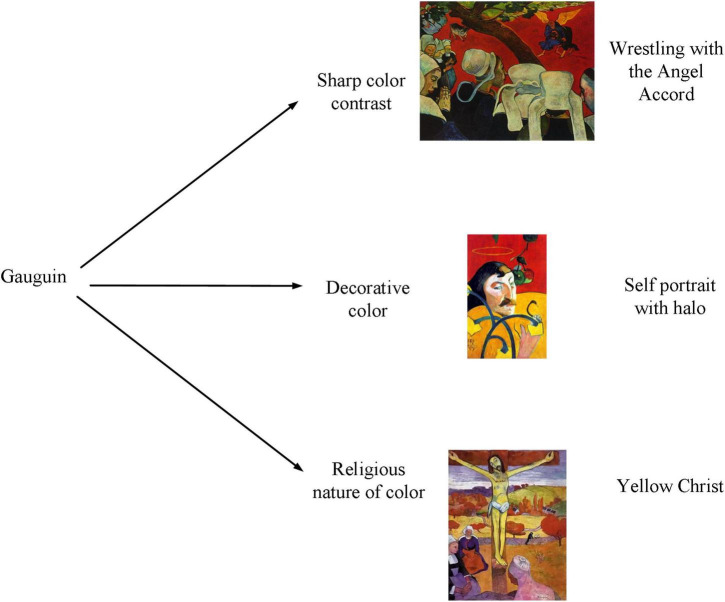
Colors in Gauguin’s paintings.

Colors in Gauguin’s paintings are shown in Figure 3.

The word “color” is an eternal topic in the field of painting. Artists can use color flexibly and skillfully to express the content of the picture when creating paintings, which shows the importance of visual color in artistic creation.

## Experiments on psychological factors and colors

The spectrum of an object determines the optical properties of the object, including its color. Different spectrums can be received by humans as the same color. Color comes from nature, and humans give it meaning. In the objective world, there are always certain colors that have had a special impact on individuals. In painting, the expression of emotion does not necessarily rely on the use of numerous colors, but lies in the control of specific color characteristics in different contexts. It is necessary to establish a set of its own color system according to its own emotions and attention to the subject.

### Factors for affecting the color tone of the picture

**FIGURE 4:**
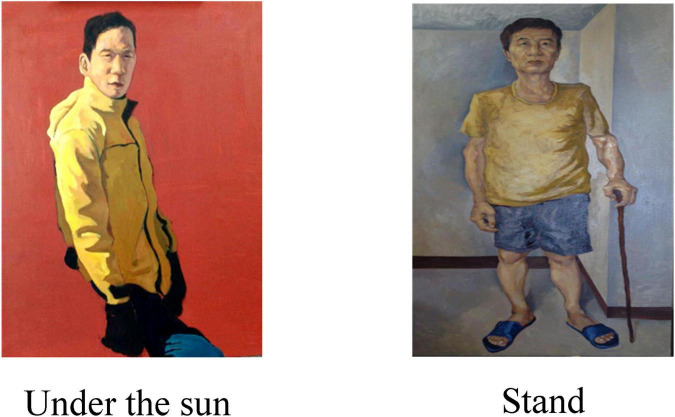
Comparison of warm and cool colors in painting.

The tendency of the overall color of a painting determines the tendency of the tones of the painting, which is the overall effect of the unity of all colors in the painting. Hue includes factors such as warmth, lightness, purity, and hue. Hue is the primary characteristic of color and the most accurate standard for distinguishing various colors. In fact, any color other than black, white and gray has the property of hue, and hue is composed of primary colors, secondary colors and complex colors. Here, two pieces of academic works from the oil painting group of a university’s art major are selected for comparison, as shown in Figure 4.

It can be seen from the Figure that different tonal tendencies make viewers have different subjective feelings in the painting, and the warm and cool colors affect people’s perception of temperature. Secondly, the warm and cool colors also have an impact on people’s sense of space. In addition, these two tones have other meanings in painting. For example, cool tones can also make people feel moist, light, quiet, low, and hard. Conversely, warm tones can feel dry, heavy, warm, soft and so on.

**FIGURE 5:**
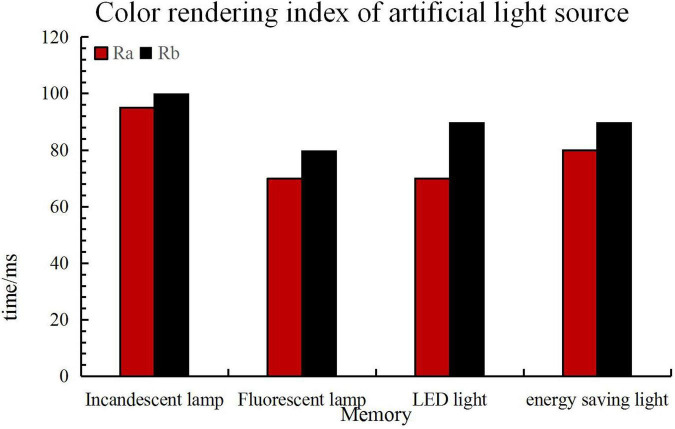
Color rendering index of common indoor artificial light sources.

The quality of lighting in a color environment is affected by both the ability to express the color and the degree of light diffusion. In artificial light sources, the index of color reproduction ability is the color rendering of the light source. Under a light source with better color rendering, the color of the object is closer to the original color of the object. The color rendering index of common indoor artificial light sources is shown in Figure 5.

In the figure, Ra is the minimum value of the color rendering index, and Rb is the maximum value of the color rendering index.

The following is a survey of the illuminance value of the painting. The paintings of the above-mentioned postgraduates majoring in painting are selected to compare the average illuminance Ai, the average illuminance Aivp of the vertical desktop of the easel, and the flat illuminance Bpi of the booth.

**FIGURE 6:**
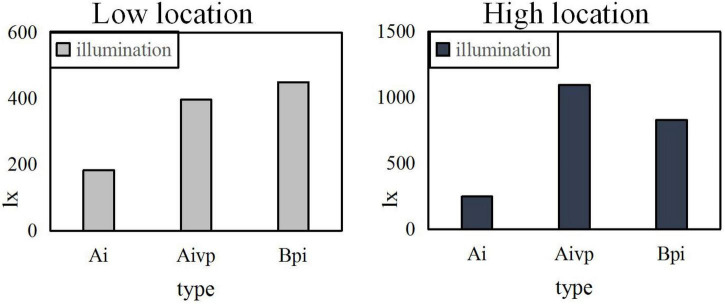
The illuminance contrast of the position when the uniformity U0 is 0.3.

When the uniformity U0 is 0.3, the same painting is placed on the high and low sides for comparison, as shown in Figure 6.

**FIGURE 7:**
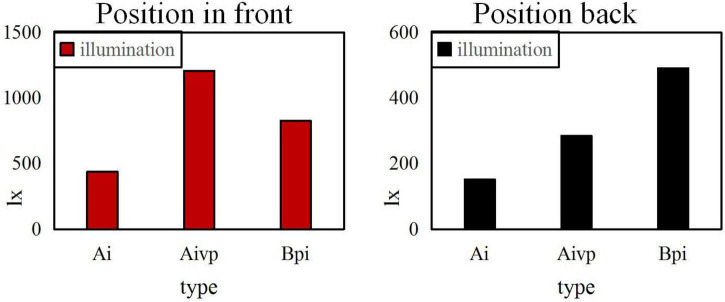
Illumination comparison before and after the position when the uniformity U0 is 0.4.

When the uniformity U0 is 0.4, the same painting is placed on the front and back sides for comparison, as shown in Figure 7.

**FIGURE 8:**
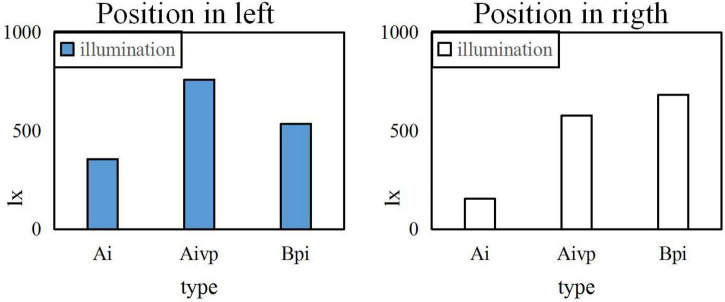
Illumination comparison before and after the position when the uniformity U0 Is 0.5.

When the uniformity U0 is 0.5, the same painting is placed on the left and right sides for comparison, as shown in Figure 8.

It can be seen from the above three Figures that the higher the uniformity U0, the better the experimental effect is. The higher and the more forward the position, the greater the impact on the level of illumination is. However, the left and right placement of the painting has little effect on the brightness. From the Figure, it can also be calculated that the standard average illuminance Ai is 300lx, the average illuminance of the vertical table top of the easel is 578xl, the flat illuminance Bpi of the booth is 1292xl, and the uniformity U0 is 0.6.

**FIGURE 9:**
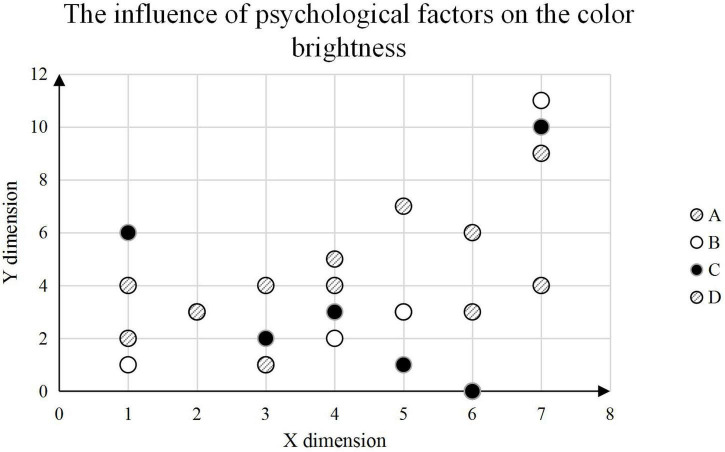
The influence of psychological factors on the judgment of color brightness.

Lightness analysis is carried out, and psychological factors are divided into four grades: A, B, C, and D. A is the happiest and D is the saddest. Rating judgments for 20 volunteers of the color environment of the same painting are collected. According to the characteristics of uniform grading of brightness, the results are classified and counted, and as shown in Figure 9.

It can be seen that among the 20 volunteers surveyed, the brightness observed by the volunteers who are happy (A) is concentrated in the range of 8–9, and more distributed between 5 and 7. Depressed (D) volunteers observed lightness centered between 3 and 5, with two appearing between 2 and 4.

### Influence of psychological factors on painting behavior

The results of correlation analysis have proved that there are some significant correlations between personality traits and three irrational behaviors: overconfidence, disposition effect and herd effect. In economics, the “herd effect” is often used to describe the herd mentality of economic individuals. The “herd effect” is a metaphor for people who have a herd mentality, which can easily lead to blind obedience, and blind obedience often leads to deception or failure. Correlation analysis can only explain the simple correlation between variables. To further study how the personality dimension interprets and predicts the dependent variable, it is necessary to carry out regression analysis to explain the strength and direction of the correlation between variables.

The correlation analysis results between the five dimensions of personality traits and the three irrational behaviors are shown in Table 1.

**TABLE 1:** Personality trait dimension-irrational behavior correlation analysis results.

		Nervous	Rigorism	Agreeableness	Openness	Extroversion
Overconfidence	Pearson correlation	−0.252*	0.438**	−0.075	0.475**	0.736**
		0.023	0.000	0.504	0.000	0.000
		81	81	81	81	81
Disposal effect	Pearson correlation	0.118	−0.176	−0.277**	0.055	0.085
		0.294	0.117	0.012	0.625	0.451
		81	81	81	81	81
Herd effect	Pearson correlation	−0.486**	0.318**	0.572**	−0.144	−0.088
		0.000	0.004	0.000	0.198	0.436
		81	81	81	81	81

The symbols * and ** refer to the magnitude of statistical difference.

Overconfidence is significantly correlated with the other four personality trait dimensions except agreeableness dimension, especially with extraversion. The disposition effect is significantly correlated with agreeableness, and the correlation strength with the five dimensions of personality traits is low. The herd effect is significantly correlated with neuroticism, conscientiousness and agreeableness, among which the correlation with agreeableness is higher.

The five dimensions of personality traits are gradually regressed to the herd effect. Through the step-by-step regression analysis, the adjusted R-square is continuously improved, agreeableness and neuroticism enter the regression equation successively, and the changes in the R-square brought by the entry of rigor, openness, and extroversion are not significantly eliminated. The regression equation is a mathematical expression that reflects the regression relationship of one variable (dependent variable) to another or a group of variables (independent variables) obtained through regression analysis based on sample data. The regression line equation is widely used, and the vector value in the regression line equation can be obtained by the least squares method to obtain the regression line equation. The results of variance analysis are shown in Table 2.

**TABLE 2:** ANOVA results.

ANOVA*						
Model		Sum of squares	Free degree	Mean square	*F*	Significance
1	Regression	206.717	1	206.717	38.451	0.000
	Residual	424.715	79	5.376		
	Amount	631.432	80			
2	Regression	235.048	2	117.524	23.126	0.000
	Residual	396.385	78	5.082		
	Amount	631.432	80			

The symbol * refers to statistical difference.

The significance test results are shown in Table 3.

**TABLE 3:** Significance test results.

Coefficient								
Model		Non-standardized coefficient		Standardization coefficient	*t*	Significance	Collinearity statistics	
			
		B	Standard error	Beta			Tolerance	VIF
1	(Constant)	−0.432	1.102		−0.392	0.696		
	Agreeableness	0.191	0.031	0.572	6.201	0.000	1.000	1.000
2	(Constant)	3.184	1.869		1.704	0.092		
	Agreeableness	0.146	0.036	0.438	4.116	0.000	0.712	1.404
	Nervous	−0.087	0.037	−0.251	−2.361	0.021	0.712	1.404

A summary of the regression model is shown in Table 4.

**TABLE 4:** Summary of regression models.

Model summary									
Model	*R*	*R* square	Adjusted R-square	Standard estimation error	R-square variation	F variation	Change statistical degrees of freedom	Freedom	Significant f change
1	0.572	0.327	0.319	2.31865	0.327	38.451	1	79	0.000
2	0.610	0.372	0.356	2.25430	0.045	5.575	1	78	0.021

After analysis of variance, the significance level of regression model 1 and model 2 reach 0.000, and the adjusted R square is 0.319 and 0.356, respectively. In this study, regression model 2 is selected as the final model. According to Model 2, the standardized regression coefficients of agreeableness and neuroticism are 0.438 and -0.251, respectively, and the regression coefficient significance is 0.000 and 0.021, respectively.

In this paper, the principal component analysis method is used to extract the common factors, and the extraction degree of each factor to the original variables is shown in Table 5.

**TABLE 5:** Common factor variance.

Variable	A1	A2	A3	A4	A5	A6	A7	A8	A9	A10	A11
Initial	1	1	1	1	1	1	1	1	1	1	1
Extract	0.612	0.836	0.741	0.812	0.764	0.779	0.734	0.815	0.67	0.741	0.702
Variable	A12	A13	A14	A15	A16	A17	A18	A19	A20	A21	–
Initial	1	1	1	1	1	1	1	1	1	1	–
Extract	0.717	0.63	0.624	0.69	0.661	0.73	0.767	0.67	0.741	0.672	–

Table 6 is the total variance explained.

**TABLE 6:** Total variance explained.

Component	Total	Total initial eigenvalue			Extract sum of squares load			Rotation sum of squares loading	
		Variance	Cumulative	Total	Variance	Cumulative	Total	Variance	Cumulative
1	5.307	32.854	32.854	5.307	32.854	32.854	3.872	23.971	23.971
2	3.503	21.684	54.538	3.503	21.684	54.538	3.263	20.202	44.174
3	2.083	12.897	67.435	2.083	12.897	67.435	2.905	17.983	62.156
4	1.107	6.851	74.287	1.107	6.851	74.287	1.631	10.096	72.251
5	1.008	6.237	80.525	1.008	3.237	80.525	1.336	8.273	80.525
6	0.837	2.740	83.265	–	–	–	–	–	–
7	0.713	1.346	84.610	–	–	–	–	–	–
8	0.688	1.270	85.880	–	–	–	–	–	–
9	0.642	1.161	87.041	–	–	–	–	–	–
10	0.621	1.147	88.188	–	–	–	–	–	–
11	0.519	1.134	89.322	–	–	–	–	–	–
12	0.505	1.123	90.445	–	–	–	–	–	–
13	0.494	1.111	91.555	–	–	–	–	–	–
14	0.463	1.099	92.654	–	–	–	–	–	–
15	0.443	1.087	93.741	–	–	–	–	–	–
16	0.420	1.075	94.816	–	–	–	–	–	–
17	0.388	1.062	95.879	–	–	–	–	–	–
18	0.358	1.050	96.928	–	–	–	–	–	–
19	0.336	1.037	97.965	–	–	–	–	–	–
20	0.309	1.024	98.990	–	–	–	–	–	–
21	0.255	1.012	100.000	–	–	–	–	–	–

The Table lists the variance contribution rate and cumulative variance contribution rate of the extracted common factors. It shows that the cumulative variance contribution rate of the first two factors among the five extracted common factors is 54.538%, and the cumulative variance contribution rate of the five common factors is 80.525%, reaching the 80% explanation level and meeting the requirements of dimensionality reduction.

## Discussion

### Forming elements of visual color

The first is that the eye itself has the ability to perceive color, which is the function of the nervous system on the retina. The second is the nature of the light source, and finally the reflection of the light on the surface of the object. All objects seen in daily life have their own color tendency. The formation of these colors is due to the reflection of the direct light from the object. That is, without the presence of light, the colors of these objects would cease to exist, and they would not be able to be seen. Therefore, it can be concluded that the color is reflected in the world because there is light. It can also be judged from this that there are two kinds of things that present color. One is the luminous body, that is, the light source. The length of the light wave determines its color, that is, the light source property factor in the visual formation of color perception factors. The other is a non-luminous body, which cannot emit light by itself, but can absorb and reflect the light of the luminous body, thereby showing color, that is, the reflection factor of the object on the light.

### The color elements of fine brushwork and heavy color painting

The research on the color elements of literati’s heavy-color painting is an important part of exploring the color integration of painting in the creation of modern literati’s heavy-color painting. First of all, it is necessary to study the development history of color application in meticulous painting, explore the characteristics of color application in meticulous painting, and analyze the inheritance and innovation of modern literati painting based on the color application of traditional literati painting. After that, it further analyzes the color application characteristics of modern literati’s heavy-color paintings, which paving the way for future comparative studies. Ink and wash paintings are mainly in black and white. In the creation of Dongba paintings, the use of two colors is relatively uniform. From the perspective of painting creation, the use of color in painting attaches great importance to the overall use of similar colors such as red and orange, so that the tone is basically unified. On this basis, colors with similar levels of cool, warm, light, and shadow are often used in combination to achieve color unity. Second, after determining the color application of the main tone, it is transferred to the details. The pinnacle of painting color lies in the pursuit of change. Uniform image colors can feel boring and aesthetically exhausting. It is the last touch to the details, which makes the color of the painting more flexible, allowing people to gain more appreciation experience and perception by appreciating the painting.

### Color vision theory

The ability of people to recognize and perceive color is called color vision, or color vision. It is the radiant energy of 380 wavelengths in the spectrum that acts on the human visual organ and produces the color perception. The process that produces color vision is universal: light travels through the cornea, daughter, lens, and vitreous to the retina, where it generates special signals, and finally reaches the brain through the optic nerve. Modern human eye color vision theory is mainly divided into two schools: Young Helmholtz’s trichromatic theory and Regin’s “opposite” color theory. According to the trichromatic theory, there are three types of nerve fibers in the human retina. Any nerve fiber produces a sense of color. These three types of nerve fibers can be called red fibers, green fibers and blue fibers. Light stimulates three primary color nerve fibers in the retina. Due to the different wavelengths, each fiber is excited to a different degree, resulting in a different perception of color. The trichromatic theory can explain the phenomenon of color mixing, but it cannot explain the phenomenon of color blindness logically.

Color can also cause different psychological changes in people. The research on the psychological effects of color is mainly divided into two parts: color perception and color preference. Color perception is the emotional changes and emotional associations that people produce after experiencing, background and visual color stimuli. This emotional change and connection is called abstract color combination. The three characteristics of color are hue, lightness and saturation that change this connection, so the emotional changes of color are very complex.

## Conclusion

In painting, the most expressive element is color. It has a strong visual effect and affects visual perception. At the same time, it can also show the inner feelings and show the various thoughts in the mind. Therefore, color is one of the most important expressions of expressionist painting. However, the human sensory system is complex and affected by multiple factors. Cognitive psychology believes that by stimulating any sense, the corresponding sensory system can be triggered. In psychology, such accompanying feeling is called “sympathy,” also known as “synaesthesia.” With the extensive attention and emphasis on human-machine cognition-related issues and human-machine design, the research on color visual comfort evaluation has achieved certain results. The article analyzes the control of line expression on the picture, discusses the forming elements of visual color, the color temptation of expressionist painting, and introduces some contents of the subject-object dichotomy. The test results showed that among the 20 volunteers surveyed, the brightness observed by the happy (A) volunteers was concentrated in the range of 8–9, and distributed more in the range of 5–7. Depressed (D) volunteers observed lightness centered between 3 and 5, with two appearing between 2 and 4. It shows that psychological factors have a great influence on the visual expression of color. It expresses the reference value and practical significance of the article in color psychology.

## Data availability statement

The original contributions presented in this study are included in the article/supplementary material, further inquiries can be directed to the corresponding author.

## Author contributions

CY and TT: writing—original draft preparation. CG, SZ, and QL: editing data curation and supervision. All authors contributed to the article and approved the submitted version.
